# Ancient DNA reveals a southern presence of the Northeast Arctic cod during the Holocene

**DOI:** 10.1098/rsbl.2022.0021

**Published:** 2022-05-04

**Authors:** Lourdes Martínez-García, Giada Ferrari, Anne Karin Hufthammer, Kjetill S. Jakobsen, Sissel Jentoft, James H. Barrett, Bastiaan Star

**Affiliations:** ^1^ Department of Biosciences, Centre for Ecological and Evolutionary Synthesis (CEES), University of Oslo, Blindernveien 31, NO-0371 Oslo, Norway; ^2^ Department of Natural HistoryThe University Museum, , University of Bergen, N-5020 Bergen, Norway; ^3^ Department of Archaeology and Cultural History, NTNU University Museum, Erling Skakkes 47b, Trondheim, Norway

**Keywords:** warm period, historical distribution, spawning distribution, feeding grounds, re-distribution, ecological interactions

## Abstract

Climate change has been implicated in an increased number of distributional shifts of marine species during the last century. Nonetheless, it is unclear whether earlier climatic fluctuations had similar impacts. We use ancient DNA to investigate the long-term spawning distribution of the Northeast Arctic cod (*skrei*) which performs yearly migrations from the Barents Sea towards spawning grounds along the Norwegian coast. The distribution of these spawning grounds has shifted northwards during the last century, which is thought to be associated with food availability and warming temperatures. We genetically identify *skrei* specimens from Ruskeneset in west Norway, an archaeological site located south of their current spawning range. Remarkably, ^14^C analyses date these specimens to the late Holocene, when temperatures were warmer than present-day conditions. Our results either suggest that temperature is not the only driver influencing the spawning distribution of Atlantic cod, or could be indicative of uncertainty in palaeoclimate reconstructions in this region. Regardless, our findings highlight the utility of aDNA to reconstruct the historical distribution of economically important fish populations and reveal the complexity of long-term ecological interactions in the marine environment.

## 1. Introduction

Significant poleward shifts in the distribution of marine species have been observed during the last century and have been associated with global warming [1]. The description of species distributions under a changing climate may yield fundamental insights into ecosystem dynamics and responses to future climate change. Nonetheless, we still have a poor understanding of the historical distribution of marine species during the late Holocene.

**Figure 1.**
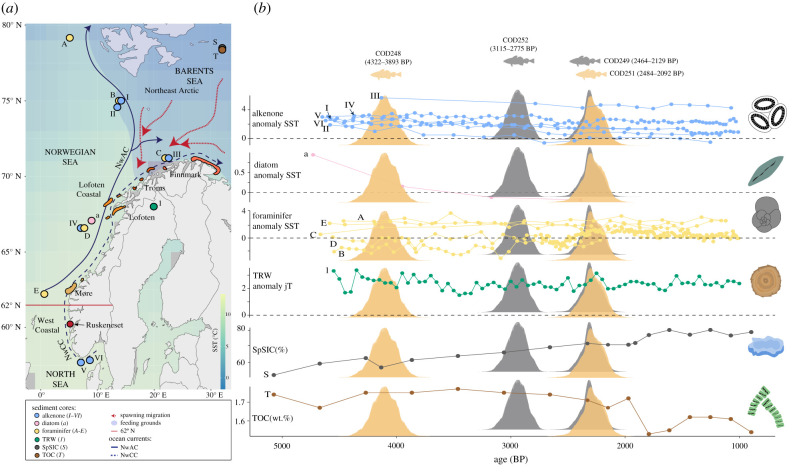
(*a*) Distribution of spawning sites for the migratory *skrei* ecotype from top to bottom: Finnmark (orange), Troms (dark orange), Lofoten (light orange) and Møre (light brown). Spawning map and details are adapted from Sundby and Nakken [4]. Blue arrows indicate the pathway of the NwAC and the NwCC. Red arrows indicate the spawning migration of *skrei* from feeding grounds. No *skrei* is currently observed below 62° N [3]. The background colour indicates average Norwegian and Barents Sea sea-surface temperature (SST) during January to December 2021. The distribution of the sediment core locations used in the study are highlighted per colour according to each proxy: alkenone (in blue: I, II, III, IV, V and VI), diatom (in pink: a), foraminifer (in yellow: A,B,C,D and E), tree-ring (TRW, in green: 1), spring sea-ice composition (SpSIC, in grey: S) and total organic carbon (TOC, in brown: T). (*b*) Historical climate reconstructions presented as an individual line for each sediment core for each proxy (electronic supplementary material, figure S2 for individual locations) with a dotted reference at 0° C in all temperature graphs. SST and July temperatures (jT) anomalies were calculated with respect to the long-term 1981–2010 average for their specific location (see electronic supplementary material, methods for details on long-term means). ^14^C dating range (orange for *skrei* and grey for stationary ecotype) are shown for each ancient Atlantic cod (see electronic supplementary material, figure S1 and table S1 for details). Specimen COD253 was not dated due to insufficient bone material. Fish illustrations were drawn by Geir Holm. Tree-ring, diatom, foraminifer, alkenone, sea ice and TOC illustrations were drawn by Lourdes Martínez-García.

Atlantic cod, an economically important and highly exploited fish species in the North Atlantic Ocean, comprises various stocks with different life-history characteristics. Along the Norwegian coast, two distinct ecological ecotypes of Atlantic cod have been identified. The ‘stationary’ ecotype (Norwegian coastal cod, NCC) spawns along the Norwegian coast and has limited migration between spawning and feeding areas [2,3]. By contrast, the ‘migratory’ ecotype (Northeast Arctic cod, NEAC), also known as ‘*skrei*’ (from the old Norse ‘the wanderer’), migrates every year during winter–spring (March to beginning of May) from colder feeding grounds (down to −1.5° C) in the Barents Sea towards warmer (up to 8° C) spawning areas along the Norwegian coast like Finnmark, Troms, Lofoten and Møre (figure 1*a*) [4,5]. In particular, the Lofoten archipelago has been the major spawning ground of *skrei* since at least medieval times, when relevant historical records first appeared [6]. Right after spawning, *skrei* eggs, larvae and juveniles will drift *ca* 600–1200 km towards the northeast of the Barents Sea, following the Norwegian Coastal Current (NwCC) and the Norwegian Atlantic Current (NwAC; figure 1*a*) [3,7].

Recent observations have shown a pronounced northward re-distribution of *skrei* [3,5]. The causes for this shift have been debated [2,4,8], although a northward movement of prey in the Barents Sea, directly influenced by an increase in sea temperatures, has been implicated [4]. Displacement of *skrei* feeding grounds lengthens migration distances to southern spawning locations (i.e. Møre) and could potentially influence the spawning latitude of *skrei* [5]. Nonetheless, it is unclear whether historical climate fluctuations along with ecological interactions (i.e. prey–predator interaction) have similarly influenced such distributions over longer temporal scales. Archaeological bone assemblages of *skrei* and the stationary ecotype are morphologically similar and difficult to distinguish with certainty [6]. Yet, the two ecotypes are genetically different, with significant population differentiation in several chromosomal inversions [6,9]. Genome-wide scans of such chromosomal regions allow the identification of individual *skrei* ecotypes with high confidence [6], even when using low-coverage sequence data from poorly preserved archaeological specimens [10].

We used ancient DNA (aDNA) to study the long-term spawning distribution of the stationary and migratory Atlantic cod ecotypes. We obtained genome-wide data of five archaeological specimens (*ca* 4322–2092 cal. BP) from Ruskeneset, west Norway (figure 1*a* and electronic supplementary material, table S1). Given the low latitudes of Ruskeneset, overall warmer climatic conditions and low sea-ice conditions in the Barents Sea during the late Holocene (within the past *ca* 5000 years, figure 1*b* and electronic supplementary material, figure S2) [11], and the contemporary temperature-related shifts in distribution, we expected that our ancient specimens would comprise stationary ecotypes.

## 2. Material and methods

### (a) Sample collection and age calibration

Ancient samples (*n* = 6) retrieved in 1914–1916 at the archaeological site Ruskeneset in the municipality of Bergen, west Norway (60.23° N – 5.15° E) [12] were used to extract DNA. The zooarchaeological assemblage (bones) from Ruskeneset are in the osteological collections at the Natural History Department, the University Museum, University of Bergen.

Ruskeneset is a rock-shelter area in the western coast of Norway which preserves evidence of human activities (e.g. bones, shells and archaeological elements) dating back to the late Neolithic and Bronze Age [13]. During the Bronze Age, the shelter would have been nearly inaccessible from land due to steep cliffs on both east and west, with easier access from the seaside by boat (N. Anfinset pers. comm.). Moreover, the fishing and hunting gear findings (e.g. harpoons, hooks, arrowheads and daggers) indicate that this was a hunting and fishing station rather than a permanent coastal settlement [13]. The site is located close to tidal current channels and is at a lower latitude than the current spawning grounds of *skrei* [3,4]. Four specimens were dated using ^14^C content (figure 1*b*; electronic supplementary material, figure S1 and table S1). Age calibration of the samples was calculated in OxCal v. 4.4.4 [14] using the Marine20 calibration curve [15]. We used slightly different ΔR values for the stationary (−164 ± 29) and *skrei* (−144 ± 46) ecotypes to account for differences in the marine reservoir effect given that these ecotypes feed either around the coast of Norway or in the Barents Sea (figure 1*b*; electronic supplementary material, figure S1) [15,16].

### (b) DNA extraction and library amplification

All ancient samples were processed in the aDNA laboratory at the University of Oslo under rigorous conditions [17,18]. DNA extraction and library preparation were according to Ferrari *et al*. [19]. Ancient read data for five specimens were processed using PALEOMIX 2.13 [20]. Sequencing reads were trimmed, filtered and collapsed using AdapterRemoval v. 2.1.7 [21], and aligned to the Atlantic cod gadMor2 nuclear genome [22,23] using BWA *backtrack* v. 0.7.12 [24] with a minimum quality score of 25. DNA postmortem damage was assessed using MapDamage v. 2.0.9 [25] and the resulting BAM files were indexed with samtools v. 1.9 [26]. Additional details of the laboratory protocols are provided in the electronic supplementary material.

### (c) Genomic statistical analyses

Four different chromosomal inversions associated with migratory behaviour and temperature clines were investigated (LG1, LG2, LG7 and LG12) to determine the probability of the ancient Atlantic cod specimens to be *skrei* [9,27–30]. These chromosomal inversions differ in their affinity towards a particular geographic area as previously described in Star *et al*. [6]. The BAMscorer pipeline [10] was used to assign inversion haplotypes. First, the Atlantic cod reference SNP database from Ferrari *et al*. [10] was used to associate divergent SNPs to different haplotypes. This reference SNP database includes 276 Atlantic cod individuals from three geographical locations (western Atlantic, eastern Atlantic and Baltic Sea) [31,32] across the species' range. Second, five ancient Atlantic cod specimens were compared to the reference dataset with *score_bams*. Ancient specimens were identified as *skrei* or stationary Lofoten Coastal or stationary (Norwegian) West Coastal individuals using the population specific chromosomal inversion frequencies obtained from Star *et al*. [6] and Johansen *et al*. [33].

### (d) Reference palaeoclimate datasets

To describe the climate as reflected during the late Holocene, particularly during the period of the Atlantic cod ancient samples (*ca* 4322–2092 cal. BP), a range of previously published marine and terrestrial palaeoreconstructions were compiled using temperature, spring sea-ice conditions (SpSIC) and total organic carbon (TOC) reconstructions along the Norwegian coast, Scandinavia and northern Barents Sea (electronic supplementary material, table S2). The localities of these datasets overlap with the spatial distribution of spawning and feeding areas of *skrei* (figure 1*a*).

Marine palaeoreconstructions are established from reference sea-surface temperature (SST) datasets based on three different proxies: alkenone (U^K’^37) [34–36], planktic foraminifer [34,37,38] and diatom assemblages [39]. Reference SpSIC dataset is based on the seasonal sea-ice biomarker IP_25_ [40], while TOC is based on the open water phytoplankton biomarkers brassicasterol and HBI III [40]. For further comparisons, SpSIC previously reported in Pieńkowski *et al*. [41] was included. This dataset includes recent observations of persisting levels of seasonal sea-ice during the Holocene Thermal Maximum (6000–10 000 cal. BP; electronic supplementary material, figure S2). The terrestrial palaeoreconstruction is established from a reference July temperature (jT) dataset based on tree-ring width (TRW) data [42]. TRW was selected because tree growth is a reliable and sensitive proxy for climatic conditions (e.g. temperatures, precipitation and drought) [43]. All temperatures are presented as an individual line for each sediment core for each proxy (figure 1*b*) and as individual graphs (electronic supplementary material, figure S2) to avoid introducing uncertainty between proxies. Full details of climate datasets are provided in the electronic supplementary material and electronic supplementary material, table S2.

## 3. Results

**Figure 2.**
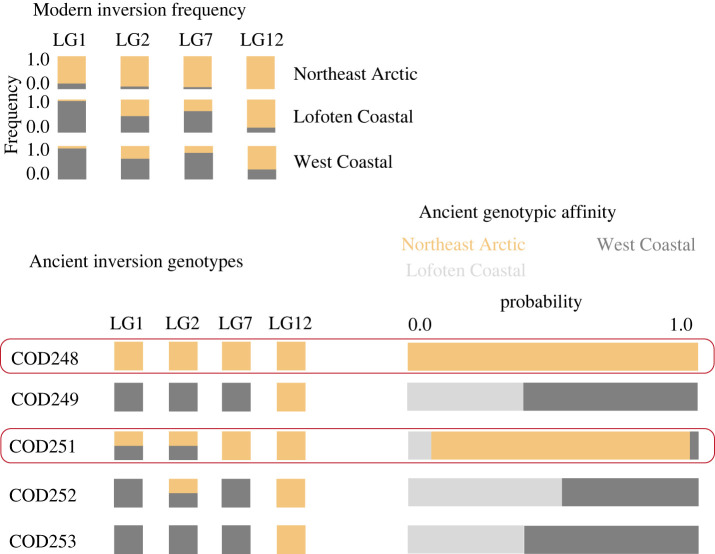
Modern inversion frequencies for LG1, LG2, LG7 and LG12 in Northeast Arctic, Lofoten Coastal and (Norwegian) West Coastal populations, and individual ancient inversion status. Binomial probability calculations identify two Atlantic cod specimens as *skrei* following Star *et al*. [6] (see electronic supplementary material, tables S3 and S4 for complete assignment probabilities).

We successfully extracted aDNA from five out of six Atlantic cod specimens and radiocarbon dated four specimens (electronic supplementary material, table S1). Sequencing reads showed the patterns of DNA fragmentation and deamination rates that are associated with authentic aDNA (electronic supplementary material, figure S3). Our sequencing results yield approximately 59 million paired reads, with between 1% and 7% endogenous DNA and approximately 74 000 to approximately 1 million aligned reads for five specimens (electronic supplementary material, table S1). This is sufficient coverage to unequivocally determine the genotype of the four major chromosomal inversions of Atlantic cod (LG1, LG2, LG7 and LG12; electronic supplementary material, table S3). Two out of five specimens (40%) were identified as *skrei* with a near 100% probability (figure 2; electronic supplementary material, tables S3 and S4). The specimens were dated to three different periods approximately 4300, approximately 3100 and approximately 2400 cal. BP which is consistent with previous dates obtained for Ruskeneset [13,44]. These specimens represent the oldest genetically identified southern *skrei* to date. Although our sample size remains limited, our findings suggest a presence of *skrei* at Ruskeneset between *ca.* 4322 and 2092 cal. BP at overall warmer temperatures than present-day conditions (figure 1*b*; electronic supplementary material, figure S2).

## 4. Discussion

Several reasons may explain the historical *skrei* presence at lower and warmer latitudes than today. First, *skrei* could have been obtained from northern latitudes and transported to Ruskeneset. Such transport would indicate the mobility of human settlements during the late Neolithic and/or Bronze Age from northern to southern Norway. Nonetheless, bone material and artefact evidence indicate that Ruskeneset was a hunting site associated with local marine exploitation [13]. Transport of *skrei* from northern Norway to this location therefore seems improbable.

Second, it is possible that the association of these inversion haplotypes with the *skrei* ecotype is of a recent evolutionary origin, and that the adaptive association we use to distinguish each ecotype has not remained stable across time. Nevertheless, the evolutionary origin of these inversions is dated to 0.4 and 1.66 million years ago, and they have been selectively maintained within the Atlantic cod populations ever since [9,30]. It would seem unlikely that the association of such evolutionary ancient genetic variants with this distinct behaviour evolved as recently as the last millennia.

Third, although no *skrei* is currently observed below 62° N (figure 1*a*), they were sporadically observed below 62° N during the start of the twentieth century [8]. The impact of fishing has been hypothesized for the current absence of such southern spawners—through the removal of larger individuals with greater capacity for migration—however, larger fish are not associated with increased migration distance [2]. The reasons for these observations, therefore, remain unknown. The archaeological southern latitude of *skrei* could reflect such sporadic spawning events, possibly during short cold spells—representing annual inter-variability—experienced within the date range of each individual and the temperature variability resolution of the palaeoclimate reconstructions (figure 1*b*; electronic supplementary material, figure S2). Moreover, the complexity of marine reservoir correction on tissues from animals feeding at latitudes higher than 50° N adds uncertainty to the precision of radiocarbon dates. Regardless, the probability of observing such sporadic southern spawning events would appear low, given that only a few specimens were sampled over a *ca* 4000 years period of natural history. Our results, therefore, tentatively suggest more frequent southern spawning of *skrei* during the late Holocene.

Finally, there may be uncertainty around the climatic reconstructions in the Barents Sea. A recent observation has identified persisting levels of seasonal sea-ice during the entire Holocene Thermal Maximum (6000–10 000 cal. BP) in this region [41] (electronic supplementary material, figure S2). Consequently, as the climate in the Barents Sea further cooled during the late Holocene (*ca* 5900 cal. BP to present) [11], this region may have had reduced primary productivity and more significant ice cover than currently estimated [40]. Such a scenario could have resulted in more southern located feeding grounds and decreasing migration distance towards lower latitude spawning areas. Our observations would agree with such more extensive presence of sea-ice than currently assumed during the late Holocene in the Barents Sea.

Taken together, we here identify the oldest known migratory ecotype in an archaeological Atlantic cod fishbone assemblage. Although the reasons for their southern distribution during the late Holocene remain unclear, our results highlight the utility of aDNA to reconstruct the historical distribution of economically important fish populations. Our findings indicate that the response of marine species to present-day and future climate change may be more complex than currently anticipated.

## Data Availability

Modern reference raw sequence data have been released earlier under ENA accession nos. PRJEB29231 and PRJEB41431. The raw reads for the ancient specimens for this study are released under ENA accession no. PRJEB49220. The data are provided in the electronic supplementary material [45].

## References

[1] Hastings RA, Rutterford LA, Freer JJ, Collins RA, Simpson SD, Genner MJ. 2020 Climate change drives poleward increases and equatorward declines in marine species. Curr. Biol. **30**, 1572-1577. (10.1016/j.cub.2020.02.043) 32220327

[2] Langangen Ø et al. 2019 Ticket to spawn: combining economic and genetic data to evaluate the effect of climate and demographic structure on spawning distribution in Atlantic cod. Glob. Change Biol. **25**, 134-143. (10.1111/gcb.14474) PMC7379705 30300937

[3] Jorde PE, Huserbråten MBO, Seliussen BB, Myksvoll MS, Vikebø FB, Dahle G, Aglen A, Johansen T. 2021 The making of a genetic cline: introgression of oceanic genes into coastal cod populations in the Northeast Atlantic. Can. J. Fish. Aquat. Sci. **78**, 958-968. (10.1139/cjfas-2020-0380)

[4] Sundby S, Nakken O. 2008 Spatial shifts in spawning habitats of Arcto-Norwegian cod related to multidecadal climate oscillations and climate change. ICES J. Mar. Sci. **65**, 953-962. (10.1093/icesjms/fsn085)

[5] Sandø A, Johansen G, Aglen A, Stiansen JE, Renner A. 2020 Climate change and new potential spawning sites for Northeast Arctic cod. Front. Mar. Sci. **7**, 28. (10.3389/fmars.2020.00028)

[6] Star B et al. 2017 Ancient DNA reveals the Arctic origin of Viking Age cod from Haithabu, Germany. Proc. Natl Acad. Sci. USA **114**, 9152-9157. (10.1073/pnas.1710186114) 28784790 PMC5576834

[7] Ottersen G, Bogstad B, Yaragina NA, Stige LC, Vikebø FB, Dalpadado P. 2014 A review of early life history dynamics of Barents Sea cod (*Gadus morhua*). ICES J. Mar. Sci. **71**, 2064-2087. (10.1093/icesjms/fsu037)

[8] Jørgensen C, Dunlop ES, Opdal AF, Fiksen Ø. 2008 The evolution of spawning migrations: state dependence and fishing-induced changes. Ecology **89**, 3436-3448. (10.1890/07-1469.1) 19137949

[9] Matschiner M et al. 2022 Supergene origin and maintenance in Atlantic cod. Nat. Ecol. Evol. 6, 469-481. (10.1038/s41559-022-01661-x) 35177802 PMC8986531

[10] Ferrari G, Atmore LM, Jentoft S, Jakobsen KS, Makowiecki D, Barrett JH, Star B. 2021 An accurate assignment test for extremely low-coverage whole-genome sequence data. Mol. Ecol. Res. **1**, 15. (10.1101/2021.06.04.447098) 34779123

[11] Wanner H et al. 2008 Mid- to Late Holocene climate change: an overview. Quat. Sci. Rev. **27**, 1791-1828. (10.1016/j.quascirev.2008.06.013)

[12] Brinkmann A, Shetelig H. 1920 III ruskenesset: en Stenalders Jaktplass (III Ruskenesset: a Stone Age Hunter Site). Christiania, Norway: A.W. Brøggers Boktrykkeri A/S.

[13] Melheim AL. 2012 Recycling ideas: bronze age metal production in southern Norway. PhD thesis, University of Oslo, Oslo, Norway.

[14] Ramsey CB. 2009 Bayesian analysis of radiocarbon dates. Radiocarbon **51**, 337-360. (10.1017/S0033822200033865)

[15] Heaton TJ et al. 2020 Marine20—the marine radiocarbon age calibration curve (0–55,000 cal BP). Radiocarbon **62**, 779-820. (10.1017/RDC.2020.68)

[16] Reimer PJ, Reimer RW. 2001 A marine reservoir correction database and on-line interface. Radiocarbon **43**, 461-463. (10.1017/S0033822200038339)

[17] Cooper A, Poinar HN. 2000 Ancient DNA: do it right or not at all. Science **289**, 1139. (10.1126/science.289.5482.1139b) 10970224

[18] Gilbert MTP, Bandelt HJ, Hofreiter M, Barnes I. 2005 Assessing ancient DNA studies. Trends Ecol. Evol. **20**, 541-544. (10.1016/j.tree.2005.07.005) 16701432

[19] Ferrari G et al. 2021 The preservation of ancient DNA in archaeological fish bone. J. Archaeol. Sci. **126**, 105317. (10.1016/j.jas.2020.105317)

[20] Schubert M et al. 2014 Characterization of ancient and modern genomes by SNP detection and phylogenomic and metagenomic analysis using PALEOMIX. Nat. Protoc. **9**, 1056. (10.1038/nprot.2014.063) 24722405

[21] Lindgreen S. 2012 AdapterRemoval: easy cleaning of next-generation sequencing reads. BMC Res. Notes **5**, 337. (10.1186/1756-0500-5-337) 22748135 PMC3532080

[22] Star B et al. 2011 The genome sequence of Atlantic cod reveals a unique immune system. Nature **477**, 207-210. (10.1038/nature10342) 21832995 PMC3537168

[23] Tørresen OK et al. 2017 An improved genome assembly uncovers prolific tandem repeats in Atlantic cod. BMC Genomics **18**, 1-23. (10.1186/s12864-016-3448-x) 28100185 PMC5241972

[24] Li H, Durbin R. 2009 Fast and accurate short read alignment with Burrows–Wheeler transform. Bioinformatics **25**, 1754-1760. (10.1093/bioinformatics/btp324) 19451168 PMC2705234

[25] Jónsson H, Ginolhac A, Schubert M, Johnson PL, Orlando L. 2013 mapDamage2. 0: fast approximate Bayesian estimates of ancient DNA damage parameters. Bioinformatics **29**, 1682-1684. (10.1093/bioinformatics/btt193) 23613487 PMC3694634

[26] Li H, Handsaker B, Wysoker A, Fennell T, Ruan J, Homer N, Marth G, Abecasis G, Durbin R. 2009 The sequence alignment/map format and SAMtools. Bioinformatics **25**, 2078-2079. (10.1093/bioinformatics/btp352) 19505943 PMC2723002

[27] Barney BT, Munkholm C, Walt DR, Palumbi SR. 2017 Highly localized divergence within supergenes in Atlantic cod (*Gadus morhua*) within the Gulf of Maine. BMC Genomics **18**, 1-14. (10.1186/s12864-017-3660-3) 28359300 PMC5374575

[28] Sodeland M et al. 2016 ‘Islands of divergence’ in the Atlantic cod genome represent polymorphic chromosomal rearrangements. Genome Biol. Evol. **8**, 1012-1022. (10.1093/gbe/evw057) 26983822 PMC4860689

[29] Berg PR, Star B, Pampoulie C, Sodeland M, Barth JM, Knutsen H, Jakobsen KS, Jentoft S. 2016 Three chromosomal rearrangements promote genomic divergence between migratory and stationary ecotypes of Atlantic cod. Sci. Rep. **6**, 1-12. (10.1038/s41598-016-0001-8) 26983361 PMC4794648

[30] Berg PR, Star B, Pampoulie C, Bradbury IR, Bentzen P, Hutchings JA, Jentoft S, Jakobsen KS. 2017 Trans-oceanic genomic divergence of Atlantic cod ecotypes is associated with large inversions. Heredity **119**, 418-428. (10.1038/hdy.2017.54) 28930288 PMC5677996

[31] Barth JMI et al. 2019 Disentangling structural genomic and behavioural barriers in a sea of connectivity. Mol. Ecol. **28**, 1394-1411. (10.1111/mec.15010) 30633410 PMC6518941

[32] Pinsky ML et al. 2021 Genomic stability through time despite decades of exploitation in cod on both sides of the Atlantic. Proc. Natl Acad. Sci. USA **118**, e2025453118. (10.1073/pnas.2025453118) 33827928 PMC8054022

[33] Johansen T, Besnier F, Quintela M, Jorde PE, Glover KA, Westgaard JI, Dahle G, Lien S, Kent MP. 2020 Genomic analysis reveals neutral and adaptive patterns that challenge the current management regime for East Atlantic cod Gadus morhua L. Evol. Appl. **13**, 2673-2688. (10.1111/eva.13070) 33294016 PMC7691467

[34] Eldevik T et al. 2014 A brief history of climate—the northern seas from the Last Glacial Maximum to global warming. Quat. Sci. Rev. **106**, 225-246. (10.1016/j.quascirev.2014.06.028)

[35] Emeis KC, Struck U, Blanz T, Kohly A, Voβ M. 2003 Salinity changes in the central Baltic Sea (NW Europe) over the last 10 000 years. The Holocene **13**, 411-421. (10.1191/0959683603hl634rp)

[36] Rigual-Hernandez AS et al. 2017 Svalbard ice-sheet decay after the Last Glacial Maximum: new insights from micropalaeontological and organic biomarker paleoceanographical reconstructions. Palaeogeogr. Palaeoclimatol. Palaeoecol. **465**, 225-236. (10.1016/j.palaeo.2016.10.034)

[37] Werner K, Müller J, Husum K, Spielhagen RF, Kandiano ES, Polyak L. 2016 Holocene sea subsurface and surface water masses in the Fram Strait—comparisons of temperature and sea-ice reconstructions. Quat. Sci. Rev. **147**, 194-209. (10.1016/j.quascirev.2015.09.007)

[38] Risebrobakken B, Dokken T, Smedsrud LH, Andersson C, Jansen E, Moros M, Ivanova EV. 2011 Early Holocene temperature variability in the Nordic Seas: the role of oceanic heat advection versus changes in orbital forcing. Paleoceanography **26**, PA4206. (10.1029/2011PA002117)

[39] Koç N, Jansen E, Haflidason H. 1993 Paleoceanographic reconstructions of surface ocean conditions in the Greenland, Iceland and Norwegian seas through the last 14 ka based on diatoms. Quat. Sci. Rev. **12**, 115-140. (10.1016/0277-3791(93)90012-B)

[40] Berben SM, Husum K, Navarro-Rodriguez A, Belt ST, Aagaard-Sørensen S. 2017 Semi-quantitative reconstruction of early to late Holocene spring and summer sea ice conditions in the northern Barents Sea. J. Q. Sci. **32**, 587-603. (10.1002/jqs.2953)

[41] Pieńkowski AJ et al. 2021 Seasonal sea ice persisted through the Holocene thermal maximum at 80°N. Commun. Earth Environ. **2**, 124. (10.1038/s43247-021-00191-x)

[42] Helama S, Seppä H, Bjune AE, Birks HJB. 2012 Fusing pollen-stratigraphic and dendroclimatic proxy data to reconstruct summer temperature variability during the past 7.5 ka in subarctic Fennoscandia. J. Paleolimnol. **48**, 275-286. (10.1007/s10933-012-9598-1)

[43] Linderholm HW, Chen D. 2005 Central Scandinavian winter precipitation variability during the past five centuries reconstructed from *Pinus sylvestris* tree rings. Boreas **34**, 43-52. (10.1111/j.1502-3885.2005.tb01003.x)

[44] Hjelle KL, Hufthammer AK, Bergsvik KA. 2006 Hesitant hunters: a review of the introduction of agriculture in western Norway. Environ. Archaeol. **11**, 147-170. (10.1179/174963106(123188)

[45] Martínez-García L, Ferrari G, Hufthammer AK, Jakobsen KS, Jentoft S, Barrett JH, Star B. 2022 Ancient DNA reveals a southern presence of the Northeast Arctic cod during the Holocene. *FigShare*. (10.6084/m9.figshare.c.5942011) PMC9065953 35506242

